# Supplementary material for: Competition and resource depletion shape the thermal response of population fitness in Aedes aegypti
Source: Commun Biol. 2022 Jan 19;5:66. doi: 10.1038/s42003-022-03030-7 (PMC8770499; doi:10.1038/s42003-022-03030-7)
Supplement: Supplementary file 2 — Supplementary Information [file 42003_2022_3030_MOESM2_ESM.pdf]

Supplementary File 1

Competition and resource depletion shape the thermal response of population fitness in *Aedes aegypti*

Supplementary Note 1

Method used to predict fecundity rate for matrix projection models (main text Equation 1)

We measured each individual's dry mass to the nearest 0.01 mg using a microbalance. Prior to weighing, mosquitoes were dried individually in microcentrifuge tubes containing desiccant-silica gel for a minimum of 14 days. For the temperature-dependent scaling between mass and wing length, we analysed the van Heuvel [1] dataset. This showed that as temperatures increase from 25 to 34°C, the scaling between mass and wing length changes significantly (Supplementary Figure 1a). Our analysis of the Farjana [2] dataset indicated that the scaling between wing length and fecundity changes significantly with temperature but not resource level (Supplementary Figure 1b).

To estimate lifetime fecundity (F in Supplementary Equation 1) from mass for mosquitoes that we reared at 22°C at all food densities, we predicted wing length from mass using the mass-to-wing length exponent at 25°C in the van Heuvel [1] dataset. We used these wing lengths to predict fecundity using the wing length-to-fecundity scaling exponent from the Farjana [2] (n = 264, R^2 = 0.87, P<0.001; Supplementary Equation 1) dataset at their at 20°C.

For mosquitoes that we reared at 26°C, there is no corresponding temperature treatment in the Farjana [2] dataset, so we first predicted wing length from mass using the mass-to-wing length exponent at 25°C in the van Heuvel[1] dataset. We then predicted fecundity using the wing length-to-fecundity scaling from the Briegel[3] dataset at 27°C (n = 206, R^2 = 0.77, P<0.001; Supplementary Equation 1). For mosquitoes that we reared at 32 and 34°C, we predicted wing length from mass using the mass-to-wing length exponent at 34°C in the in the van Heuvel[1] dataset. We then predicted fecundity for these mosquitoes using the wing length-to-fecundity scaling exponent from the Farjana [2] (Supplementary Equation 1) dataset at 30°C. Fecundity was not estimated at 36°C, as no adults emerged at this temperature. The **scaling equations used to estimate temperature-dependent fecundity from wing length for our mosquitoes are:**

$$\begin{aligned} &22°C,\ F = 0.93 + 3.16 \log(L) \\ &26°C,\ F = 0.40 + 3.80 \log(L) \\ &32°C,\ F = 0.26 + 4.08 \log(L) \\ &34°C,\ F = 0.26 + 4.08 \log(L) \end{aligned} \qquad \text{(Supplementary Equation 1)}$$

The coefficients were derived from our analysis (Supplementary Figure 1) of the Farjana [2] and Briegel [3] datasets.

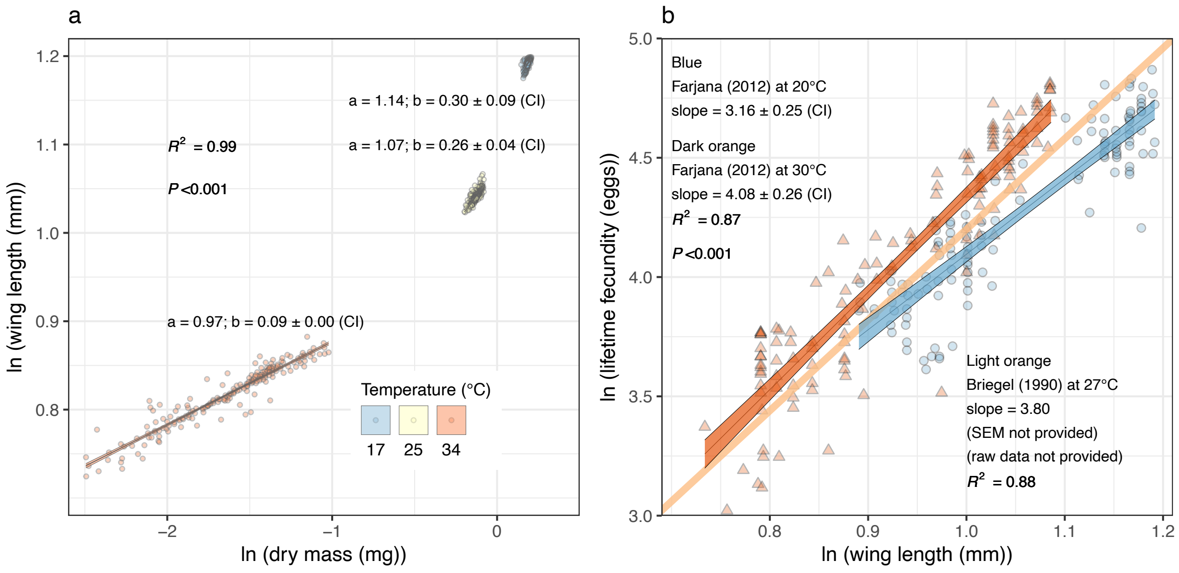

Supplementary Figure 1. **a** Analysis of the van Heuvel [1] dataset shows that the scaling of mass and wing length in *Ae. Aegypti* is temperature-dependent. The scaling exponents (slopes) for 17°C and 25°C are significantly higher than at 34°C. However, the higher scaling exponent for 17°C is non-significantly higher than for 25°C. **b** Analysis of the Farjana [2] dataset shows that the scaling of wing length and fecundity in *Ae. Aegypti* is temperature-dependent. The scaling exponents (slopes) for both resource levels are significantly higher at 30°C than at 20°C. However, the effect of resource on fecundity is non-significant at the temperature level (not shown). The standard error for the scaling exponent at 27°C is not shown because it is not provided in [3], so for 26°C, we assumed a similar 95% CI to those in the Farjana [2] dataset (3.80 ± 0.25). Despite these assumptions relating to fecundity, our r_m calculations are robust to uncertainty/variation in the underlying scaling and temperature dependencies (Figure 4).

Model terms	Model name	AIC	ΔAIC	df
**Temperature × RL**	**Interaction**	**6446.77**	**0**	**20**
Temperature × RL + replicate + block	Maximal	6450.89	+4.12	23
Temperature × RL + replicate	No block	6448.89	+2.13	22
Temperature + RL	No interaction	6462.33	+15.56	8
Temperature	Temperature only	6481.32	+34.55	5
Resource	Resource only	6899.22	+452.46	4
None	Null	6906.62	+459.86	1

Supplementary Table 1. Simplification of the exponential juvenile survival model. The maximal model includes the effects of temperature × resource level (RL) + replicate + block on mortality. The final mortality model was obtained by dropping terms from the maximal model. If removing a term worsened model fit (ΔAIC > –2), then it was retained. Otherwise, it was removed. ΔAICs were calculated as differences from the interaction model (bold).

Resource level (mg ml^{-1})	Model name	AIC	df
0.183	Kamykowski [4]	–44.43	10
0.183	Lactin2 [5]	–42.77	11
0.367	Kamykowski [4]	–65.53	10
0.367	Lactin2 [5]	–67.77	11
0.550	Kamykowski [4]	–61.31	10
0.550	Lactin2 [5]	–63.61	11
0.733	Kamykowski [4]	–53.82	10
0.733	Lactin2 [5]	–56.40	11

Supplementary Table 2. Comparison of model fitting for r_m TPCs by resource level. We considered several models that allow for negative values at both cold and hot extremes, including polynomial regression models (quadratic models underfitted the matrix projection r_m estimates, whereas cubic models overfitted these estimates (not shown) and other TPC models (not shown) that are implemented in the rTPC [6] R package. Overall, the Lactin2 [5] function and Kamykowski [4] model best described the matrix projection estimates according to the Akaike Information Criterion (AIC). Although these models performed similarly according to their AICs, we chose the Kamykowski [4] model because it was better at describing the estimated r_m at our lowest resource level.

Supplementary References

1. van den Heuvel, M. J. The Effect of Rearing Temperature on the Wing Length, Thorax Length, Leg Length and Ovariole Number of the Adult Mosquito, Aedes Aegypti (L.). *Transactions of the Royal Entomological Society of London* **115**, 197–216 (1963).

2. Farjana, T. & Tuno, N. Effect of body size on multiple blood feeding and egg retention of Aedes aegypti (L.) and Aedes albopictus (Skuse) (Diptera: Culicidae). *Medical Entomology and Zoology* **63**, 123–131 (2012).

3. Briegel, H. Metabolic relationship between female body size, reserves, and fecundity of Aedes aegypti. *Journal of Insect Physiology* **36**, 165–172 (1990).

4. Kamykowski, D. & McCollum, S. A. The temperature acclimatized swimming speed of selected marine dinoflagellates. *Journal of Plankton Research* **8**, 275–287 (1986).

5. Lactin, D. J., Holliday, N. J., Johnson, D. L. & Craigen, R. Improved Rate Model of Temperature-Dependent Development by Arthropods. *Environmental Entomology* **24**, 68–75 (1995).

6. Padfield, D., O'Sullivan, H. & Pawar, S. rTPC and nls.multstart : A new pipeline to fit thermal performance curves in R. *Methods in Ecology and Evolution* **12**, 1138–1143 (2021).
