# Supplementary material for: Competition and resource depletion shape the thermal response of population fitness in Aedes aegypti
Source: Commun Biol. 2022 Jan 19;5:66. doi: 10.1038/s42003-022-03030-7 (PMC8770499; doi:10.1038/s42003-022-03030-7)
Supplement: Supplementary file 3 — Description of Additional Supplementary Files [file 42003_2022_3030_MOESM3_ESM.pdf]

Description of Additional Supplementary Data

File name: Supplementary Data 1

Description: Contains the source data for Figure 1, and Tables 1 and 2.

File name: Supplementary Data 2

Description: Contains the source data for Figure 2 and Table 2.

File name: Supplementary Data 3

Description: Contains the source data for Figure 3.

File name: Supplementary Data 4

Description: Contains the source data for Figure 4.
