# Supplementary material for: Competition and resource depletion shape the thermal response of population fitness in Aedes aegypti
Source: Commun Biol. 2022 Jan 19;5:66. doi: 10.1038/s42003-022-03030-7 (PMC8770499; doi:10.1038/s42003-022-03030-7)
Supplement: Supplementary file 8 — Reporting Summary [file 42003_2022_3030_MOESM8_ESM.pdf]

nature portfolio

Corresponding author(s): Paul Huxley

Last updated by author(s): Dec 14, 2021

Reporting Summary

Nature Portfolio wishes to improve the reproducibility of the work that we publish. This form provides structure for consistency and transparency in reporting. For further information on Nature Portfolio policies, see our Editorial Policies and the Editorial Policy Checklist.

Statistics

For all statistical analyses, confirm that the following items are present in the figure legend, table legend, main text, or Methods section.

n/a	Confirmed	
☐	☒	The exact sample size (n) for each experimental group/condition, given as a discrete number and unit of measurement
☐	☒	A statement on whether measurements were taken from distinct samples or whether the same sample was measured repeatedly
☐	☒	The statistical test(s) used AND whether they are one- or two-sided<br>*Only common tests should be described solely by name; describe more complex techniques in the Methods section.*
☐	☒	A description of all covariates tested
☐	☒	A description of any assumptions or corrections, such as tests of normality and adjustment for multiple comparisons
☐	☒	A full description of the statistical parameters including central tendency (e.g. means) or other basic estimates (e.g. regression coefficient) AND variation (e.g. standard deviation) or associated estimates of uncertainty (e.g. confidence intervals)
☐	☒	For null hypothesis testing, the test statistic (e.g. F, t, r) with confidence intervals, effect sizes, degrees of freedom and P value noted<br>*Give P values as exact values whenever suitable.*
☒	☐	For Bayesian analysis, information on the choice of priors and Markov chain Monte Carlo settings
☒	☐	For hierarchical and complex designs, identification of the appropriate level for tests and full reporting of outcomes
☐	☒	Estimates of effect sizes (e.g. Cohen's d, Pearson's r), indicating how they were calculated

Our web collection on statistics for biologists contains articles on many of the points above.

Software and code

Policy information about availability of computer code

Data collection	*Provide a description of all commercial, open source and custom code used to collect the data in this study, specifying the version used OR state that no software was used.*
Data analysis	RStudio: Integrated Development Environment for R. Boston, MA: RStudio, Inc.; 2016

For manuscripts utilizing custom algorithms or software that are central to the research but not yet described in published literature, software must be made available to editors and reviewers. We strongly encourage code deposition in a community repository (e.g. GitHub). See the Nature Portfolio guidelines for submitting code & software for further information.

Data

Policy information about availability of data

All manuscripts must include a data availability statement. This statement should provide the following information, where applicable:

- Accession codes, unique identifiers, or web links for publicly available datasets
- A description of any restrictions on data availability
- For clinical datasets or third party data, please ensure that the statement adheres to our policy

The datasets generated and analysed during the current study are available in Supplementary Data files 1 to 4.

Field-specific reporting

Please select the one below that is the best fit for your research. If you are not sure, read the appropriate sections before making your selection.

☐ Life sciences ☐ Behavioural & social sciences ☒ Ecological, evolutionary & environmental sciences

For a reference copy of the document with all sections, see nature.com/documents/nr-reporting-summary-flat.pdf

Ecological, evolutionary & environmental sciences study design

All studies must disclose on these points even when the disclosure is negative.

Study description	In this study, we used laboratory experiments on the mosquito Aedes aegypti, combined with a stage-structured population model, to show that intensified larval competition in ecologically-realistic depleting resource environments can profoundly affect the thermal response of arthropod population fitness.
Research sample	The study was carried out on Aedes aegypti - an important vector of several emerging pathogenic arboviruses including dengue, chikungunya, and Zika viruses. We investigated the effect of larval competition in depleting resource environments on the shape of the thermal response of the vector's population fitness.
Sampling strategy	We used the results from a preliminary assay that was conduced for a previous related study to determine the adequacy of this replication level to detect statistically significant effect sizes (Electronic Supplementary Material of doi: 0.1098/rspb.2020.3217). The tubs and samples sizes (i.e. individuals per ml) also simulate a scenario commonly found in natural habitats.
Data collection	All data were collected by the first author on the manuscript. Data collection was performed using observational data collection (competition assay), instrument based data collection (Mettler Toledo™ Excellence XS Microbalance; dry mass measurements).
Timing and spatial scale	Data collection began in August 2019 and ended November 2019. Assays were performed in two randomised blocks. Each block consisted of all five temperatures and two of the resource levels.
Data exclusions	No data were excluded.
Reproducibility	All assays were performed in biological replicates and analysed using technical replicates.
Randomization	Mosquitoes were allocated into treatment groups. Allocation for biological repeats was random.
Blinding	Blinding was not possible as all experiments were carried out by only one person.

Did the study involve field work? ☐ Yes ☒ No

Reporting for specific materials, systems and methods

We require information from authors about some types of materials, experimental systems and methods used in many studies. Here, indicate whether each material, system or method listed is relevant to your study. If you are not sure if a list item applies to your research, read the appropriate section before selecting a response.

Materials & experimental systems

n/a	Involved in the study	
☒	☐	Antibodies
☒	☐	Eukaryotic cell lines
☒	☐	Palaeontology and archaeology
☐	☒	Animals and other organisms
☒	☐	Human research participants
☒	☐	Clinical data
☒	☐	Dual use research of concern

Methods

n/a	Involved in the study	
☒	☐	ChIP-seq
☒	☐	Flow cytometry
☒	☐	MRI-based neuroimaging

Animals and other organisms

Policy information about studies involving animals; ARRIVE guidelines recommended for reporting animal research

Laboratory animals	Aedes aegypti (F16-19) originating from Fort Meyer, FL, USA.
Wild animals	The study did not involve wild animals.
Field-collected samples	The study did not involve animals collected in the field.

Ethics oversight	No ethical approval required.

Note that full information on the approval of the study protocol must also be provided in the manuscript.
